# Electroacupuncture Pretreatment Alleviates LPS-Induced Acute Respiratory Distress Syndrome via Regulating the PPAR Gamma/NF-Kappa B Signaling Pathway

**DOI:** 10.1155/2020/4594631

**Published:** 2020-07-22

**Authors:** Di Feng, Huanping Zhou, Xiaohong Jin, Juan Wei, Qingqing Zhang, Yang Gu, Pengcheng Zhang, Hao Yang, Jiangang Song, Xuan Shi, Xin Lv

**Affiliations:** ^1^Department of Anesthesiology, Shanghai Pulmonary Hospital, Tongji University School of Medicine, Shanghai 200433, China; ^2^Department of Anesthesiology, The Second Affiliated Hospital of Nanchang University, Nanchang 330006, China; ^3^Department of Anesthesiology, The First Hospital of Anhui Medical University, Hefei 230022, China; ^4^Acupuncture and Anesthesia Research Institute, Shuguang Hospital Affiliated to Shanghai University of Traditional Chinese Medicine, Shanghai 200433, China

## Abstract

Electroacupuncture (EA) is reported to possess anti-inflammatory properties and has beneficial effects on acute respiratory distress syndrome (ARDS). However, the underlying mechanisms of the effects of EA on ARDS remain unclear. This study aims to investigate the protective effect of EA on LPS-induced ARDS. In this study, Sprague-Dawley male rats were treated with EA at Hegu (LI4) for 45 minutes before LPS instillation (0.4 mg/kg, 100 ul). H&E staining, wet-to-dry weight (W/D) ratio, PaO_2_, and protein content in BALF were employed to determine the function of lung tissues. Inflammatory cytokines in serum and BALF were detected by enzyme-linked immunoassay assay (ELISA). The levels of oxidative stress markers were detected to determine the oxidative stress status. Cell apoptosis was observed by terminal deoxynucleotidyl transferase-mediated dUTP nick-end labeling (TUNEL) staining and western blot. Here, we found that EA pretreatment effectively alleviated lung pathological damage. Moreover, EA suppressed the oxidative stress damage by upregulating glutathione and superoxide dismutase and downregulating malondialdehyde. EA pretreatment also regulated apoptosis-related proteins, such as Bax and Bcl-2. We found that peroxisome proliferators-activated receptors *γ* (PPAR*γ*) play a critical role during ARDS, EA up-regulated the expression of PPAR*γ*, which inhibited the activation of nuclear factor-kappa B (NF-*κ*B) and decreased the inflammatory cytokines (interleukin-1*β*, interleukin-6, and tumor necrosis factor-*α*). When rats were treated with GW9662, a selective PPAR*γ* antagonist, these effects of EA were reversed. Our study demonstrated that EA pretreatment had a beneficial effect on LPS-induced ARDS in rats by anti-inflammatory, antioxidative, and antiapoptotic properties which was regulated via PPAR*γ*/NF-*κ*B signaling pathway.

## 1. Introduction

Acute respiratory distress syndrome (ARDS) is a life-threatening form of respiratory failure. Globally, ARDS accounts for 10% of intensive care unit admissions, representing more than 3 million patients annually [1]. ARDS is characterized by noncardiogenic pulmonary edema, life-threatening hypoxemia, oxidative stress, and imbalance of proinflammatory and anti-inflammatory cytokines [2]. The LUNG SAFE study reported that the mortality rate of ARDS was 34% to 45% [3]. However, there is still no effective therapy for this life-threatening disorder because of the limited understanding about its pathogenesis.

Acupuncture is a popular folk practice in traditional Chinese medicine (TCM) with a history over 5000 years. In Europe and the USA, this traditional Chinese medicine has become one of the treatment strategies and has steadily claimed its usefulness in complementary medicine [4]. Electroacupuncture (EA), a kind of therapy, combines traditional Chinese medicine with modern technique. Increasing numbers of studies have shown that EA has an effect against inflammation. Our previous study [5] found that EA performed at the acupoints Hegu (LI-4, located at the junction of the first and second metacarpal bones) and Neiguan (PC-6, located in the groove caudal to the flexor carpi radialis and cranial to the superficial digital flexor muscles) could inhibit systemic inflammatory response in rats with lethal endotoxemia. Yang et al. [6] reported that acupuncture performed at the acupoints Dazhui (GV14, on the posterior median line in the depression below the spinous process of the seventh cervical vertebra), Fengmen (BL12, 1.5 cun lateral to the lower border of the spinous process of the second thoracic vertebra), and Feishu (BL13, 1.5 cun lateral to the lower border of the spinous process of the third thoracic vertebra) could exert a regulatory effect on mucosal and cellular immunity in patients with allergic asthma. Rafael Torres-Rosas et al. [7] found that EA at acupoint Zusanli (ST36, 2 mm lateral to the anterior tubercle of the tibia in the anterior tibial muscle and 4 mm distal to the knee joint lower point) could reduce the lipopolysaccharide- (LPS-) induced serum level of many cytokines. Other studies have explored the relationship between EA and peroxisome proliferators-activated receptors *γ* (PPAR*γ*) in patients with Alzheimer's disease (AD) and type 2 diabetes mellitus (DM2) [8, 9]; thus, it is important to explore the role of PPAR*γ* in the process of ARDS and its crucial role in the EA treatment of ARDS.

PPAR*γ* is a member of ligand-activated transcription factors superfamily PPARs. In the process of lipid metabolism homeostasis, energy homeostasis, and inflammatory response, PPAR*γ* plays a crucial role. Our previous study has shown that PPAR*γ* is one of the key mediators to alleviate Ali. [10], and other investigators also demonstrated that PPAR*γ* plays a critical role in resisting oxidant-induced lung injury [11, 12], reducing inflammation [13] and alleviating pulmonary fibrosis [14] in both mice and humans, suggesting that PPAR*γ* may be a potential therapeutic target for ARDS.

Based on these data, we hypothesized that EA at specific acupoint Hegu (LI-4) could activate PPAR*γ* and inhibit inflammatory cytokines and the inflammatory response in a rat model of ARDS.

## 2. Materials and Methods

### 2.1. Animals and Grouping

Male Sprague-Dawley (SD) rats weighing 200–300 g (SLAC Laboratory Animal Co., Ltd., Shanghai, China) were raised in a specific pathogen-free environment for a week in a 12-hour light/dark cycle, constant temperature, and humidity condition with free access to standard rodent diet and water. All animal experiments were approved by the Animal Experiment Administration Committee of the Shanghai Pulmonary Hospital and carried out in accordance with the Guide for the Care and Use of Laboratory Animals of the National Institutes of Health (NIH publications No. 8023, revised 1978). Fifty rats were equally randomized into five groups: negative control (NC) group, where the rats received intratracheal instillation of 100 ul saline by a MicroSprayer syringe assembly (MSA-250-M, Penn Century, USA) according to the previous study [15] and EA pretreatment at nonacupoint for 45 minutes; LPS group, where the rats received intratracheal instillation of lipopolysaccharide (LPS; L2630, Sigma MO, USA, 0.4 mg/kg, 100 ul) by a MicroSprayer syringe assembly and EA pretreatment at nonacupoint for 45 minutes; EA group, where the rats received intratracheal instillation of LPS by a MicroSprayer syringe assembly and 45-minute EA pretreatment at Hegu; R + EA group, where the rats received caudal vein injection of 0.3 mg/kg rosiglitazone (R), followed by 45-minute EA pretreatment at Hegu, then received intratracheal instillation of LPS by a MicroSprayer syringe assembly; and G + EA group, where the rats received caudal vein injection of 0.3 mg/kg GW9662 (G), followed by 45-minute EA pretreatment at Hegu and then received intratracheal instillation of LPS by a MicroSprayer syringe assembly.

### 2.2. Electroacupuncture Pretreatment

EA pretreatment was performed as previously described [4] at Hegu (LI4) acupoint, which is located at the junction of the first and the second metacarpal bones. A set of nonacupoints located on the ulna side of the metacarpus served as controls. All rats were anesthetized with intraperitoneal 3% 1 ml/kg pentobarbital sodium. After successful induction of anesthesia, stainless needles were inserted into bilateral acupoints of Hegu (LI4) to a depth of 5 mm, fixed by tapes. Stimulation with 4 mA current at a frequency of 2/100 Hz for 45 minutes was delivered using an EA treatment instrument (HANS LH-202, Huawei Co., Beijing, China).

### 2.3. Histopathology and Immunohistochemistry Analysis

24 hours after LPS instillation, the right upper lungs in the five groups were excised and fixed in 10% PBS buffered formalin for 24 hours at room temperature, embedded in paraffin, cut into 5-*μ*m sections, and then stained with hematoxylin and eosin (H&E) using the standard protocol. For immunohistochemistry of PPAR*γ*, Bax, and Bcl-2, the tissue sections were incubated with the primary antibody against PPAR*γ* (1 : 500, #2435, Cell Signaling Technology, Boston, USA), Bax (1 : 400, #14796, Cell Signaling Technology, Boston, USA), and Bcl-2 (1 : 500, ab182858, Abcam, Cambridge, United Kingdom). After these procedures, sections were washed with PBS three times, followed by the secondary goat antirabbit antibody (Sangon Biotech, Shanghai, China) at room temperature for 1 hour. At last, the lung histopathology and immunohistochemistry analysis were performed by pathologists blinded to experimental grouping using a digital camera (Nikon, Tokyo, Japan).

The severity of lung injury was scored based on the following histologic features as described previously [16]: (a) alveolar congestion, (b) hemorrhage, (c) infiltration or aggregation of neutrophils in the airspace or vessel wall, and (d) thickness of the alveolar wall/hyaline membrane formation. Each item was scored on a 5-point scale as follows: 0 = minimal damage, 1+ = mild damage, 2+ = moderate damage, 3+ = severe damage, and 4+ = maximal damage. The scores were summed to represent the lung injury score (total, 0–16).

### 2.4. Measurement of PaO_2_, the Lung Wet-to-Dry Weight (W/D) Ratio and Protein Content in Bronchoalveolar Lavage Fluid (BALF)

Four hours after LPS instillation, arterial blood (0.5 ml) was drawn through the common carotid artery and analyzed by Stat Profile pHOx (Nova Biomedical, Waltham, MA, USA). Then, the inferior lobe of the right lung was excised and the wet weight was recorded. The lung tissues were dried in a 60°C oven for a week and then the dry weight was recorded. The lung wet-to-dry weight (W/D) ratio was calculated to assess pulmonary edema. The protein content in BALF was measured with a BCA Protein Assay Kit (Thermo Fisher Scientific, Inc.).

### 2.5. Inflammatory Cytokines Assay

Four hours after LPS instillation, the serum, BALF, and tissue samples were collected to measure the levels of IL-1*β*, IL-6, TNF-*α*, and myeloperoxidase (MPO) via commercial enzyme-linked immunosorbent assay (ELISA) kits (BioLegend, San Diego, CA) following the protocols supplied by manufacturers.

### 2.6. Reactive Oxidative Stress Activity Assay

The levels of malondialdehyde (MDA) and glutathione (GSH) and the activities of superoxide dismutase (SOD) in the rat lung tissues were measured by commercial biochemical kits (Jiancheng Institute of Biotechnology, Nanjing, China) following the protocols supplied by manufacturers.

### 2.7. Western Blot Analysis

Western blot analysis was performed as previously described [10]. The lung tissues of the rats were prepared 4 hours after LPS instillation; the protein concentrations of all samples were determined using a BCA Protein Assay Kit (Thermo Fisher Scientific, Inc.). Total protein (30 *μ*g) was separated by 10% SDS-PAGE gel and then transferred to a polyvinylidene fluoride membrane (BioRad, Hercules, California, USA). The membrane was blocked with 5% nonfat dry milk for 2 hours at room temperature, followed by incubation with a primary antibody. The antibodies used for western blotting were as follows: *β*-actin (1 : 1000, 20536-1-AP, Proteintech, Chicago, USA), PPAR*γ* (1 : 1000, #2435, Cell Signaling Technology, Boston, USA), Bax (1 : 1000, #14796, Cell Signaling Technology, Boston, USA), Bcl-2 (1 : 1000, ab182858, Abcam, Cambridge, United Kingdom), p-p65 (1 : 1000, #3033, Cell Signaling Technology, Boston, USA), I*κ*B*α* (1 : 1000, #4814, Cell Signaling Technology, Boston, USA), and p-I*κ*B*α* (1 : 1000, #2859, Cell Signaling Technology, Boston, USA). After being washed, the membrane was incubated with the secondary antibody (1 : 5000) for 1 hour at room temperature, washed with TBST, and detected using an ECL chemiluminescence detection kit (Beyotime, P0018A, Shanghai, China) and Quantity One software (Bio-Rad). The density of each specific band was quantified with Image J.

### 2.8. TUNEL Staining of the Lung

Apoptosis in the lung tissues was assessed by terminal deoxynucleotidyl transferase-mediated dUTP nick-end labeling (TUNEL) using a commercial kit as previously described [17]. Under experimental conditions, lung tissues were fixed in 10% PBS buffered formalin for 25 minutes at 4°C and washed by PBS. 2% Triton X-100 was used to permeabilize. Lung tissues were incubated at 37°C for 60 minutes in equilibration buffer, 2-deoxynucleotide 5′-triphosphate, and terminal deoxynucleotidyl transferase (TdT) enzyme as per manufacturer's protocol. Then samples were washed in PBS. The sections were observed with a fluorescence microscope, and representative fields were chosen for application.

### 2.9. Statistical Analysis

All results are presented as the mean ± standard error of the mean (SEM) *n* = 10 rats/group. The differences between groups were determined using one-way ANOVA with Bonferroni post hoc multiple comparisons test. *P* values less than 0.05 were considered statistically significant. Statistical analyses were performed with Graph Pad Prism 7 software (La Jolla, CA, USA).

## 3. Results

### 3.1. EA Pretreatment Increases the Expression of PPAR*γ* after LPS Instillation

**Figure 1:**
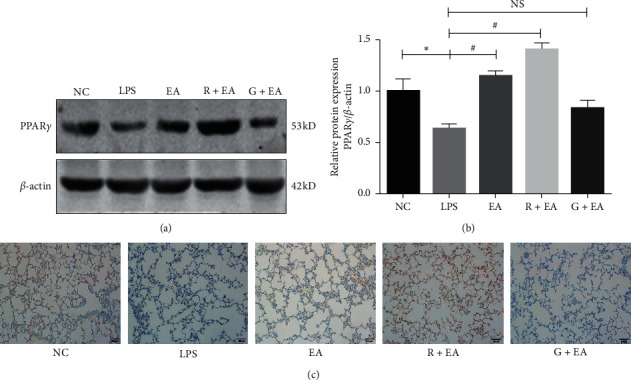
EA pretreatment increases the expression of PPAR*γ* after LPS instillation. (a) Lung tissues from rats were analyzed by western blot analysis to detect the expression of PPAR*γ*; (b) density of PPAR*γ* protein relative to that of *β*-actin; (c) immunohistochemistry analysis for PPAR*γ* in the rat lung tissues (200x). Data are presented as means ± SEM. ^*∗*^*P* < 0.05 versus NC group and ^#^*P* < 0.05 versus LPS group.

The expression of PPAR*γ* in the rat lung tissues was detected by western blot analysis at 4 hours after LPS instillation. Compared with NC group, LPS instillation decreased the expression of PPAR*γ*, while EA pretreatment upregulated the expression of PPAR*γ*. In the R + EA group, the expression of PPAR*γ* was upregulated, while treated with GW9662, the expression was decreased significantly (Figures 1(a) and 1(b)).

Levels of PPAR*γ* expression were also detected by immunohistochemistry (IHC). In NC group, the expression of PPAR*γ* was abundant and after LPS instillation, the expression of PPAR*γ* was significantly decreased. When pretreated with EA, the downregulation of PPAR*γ* was prevented in the rat lung tissues. The results in IHC were similar to that detected by western blot, in the R + EA group, the expression of PPAR*γ* was upregulated and, in G + EA group, the expression of PPAR*γ* was decreased (Figure 1(c)). These results indicated that EA pretreatment could alleviate the downregulation of PPAR*γ* in the rat lung tissues after LPS instillation.

### 3.2. EA Pretreatment Alleviates Lung Tissue Injury after LPS Instillation

**Figure 2:**
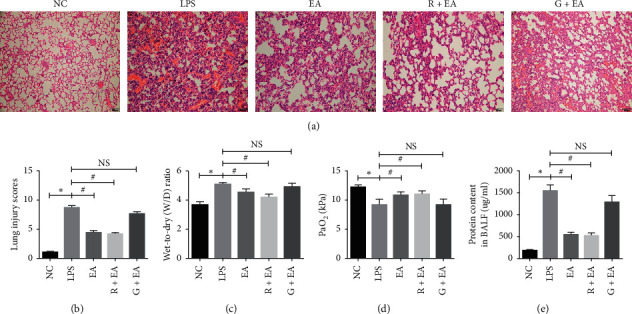
EA pretreatment alleviates lung tissue injury after LPS instillation. (a) and (b) H&E staining and lung injury score analysis of lung tissues from NC group, LPS group, EA group, R + EA group, and G + EA group (100x); (c) W/D ratios of different groups; (d) PaO_2_ analysis of different groups; (e) protein content in BALF of different groups. Data are presented as mean ± SEM. ^*∗*^*P* < 0.05 versus NC group, ^#^*P* < 0.05 versus LPS group.

Because of the changes of PPAR*γ* expression in the above three groups, the agonist and inhibitor of PPAR*γ*, rosiglitazone and GW9662, were used in R + EA group and G + EA group, respectively, before EA treatment and LPS instillation as previously described. The protective effect of EA on the rat lung tissues was evaluated by microscopy. Compared with NC group, acute inflammatory response was observed in the rat lung tissues in LPS group, as represented by the damaged thick alveolar wall, the increased number of inflammatory cells, and the increased amount of vascular congestion (Figure 2(a)). It is noteworthy that EA pretreatment attenuated the pathological changes in the rat lung tissues (Figure 2(b)).

The protein content in BALF, the wet-to-dry weight (W/D) ratio of the rat lung tissues and arterial partial pressure of oxygen (PaO_2_) were also measured to analyze the lung damage. As can be seen in Figure 2(c), W/D in LPS group was higher than that in NC group, while there was a decrease in W/D in EA and R + EA groups. Measurements of PaO_2_ in the five groups indicated that EA relieved the respiratory injury induced by LPS instillation (Figure 2(d)). In addition, the protein content in BALF showed the similar trends (Figure 2(e)). All these results suggested that EA pretreatment effectively relieved ARDS, and the effect of EA could be reversed by PPAR*γ* antagonist GW9662.

### 3.3. EA Pretreatment Suppresses the Inflammatory Response and Oxidative Stress Damage

**Figure 3:**
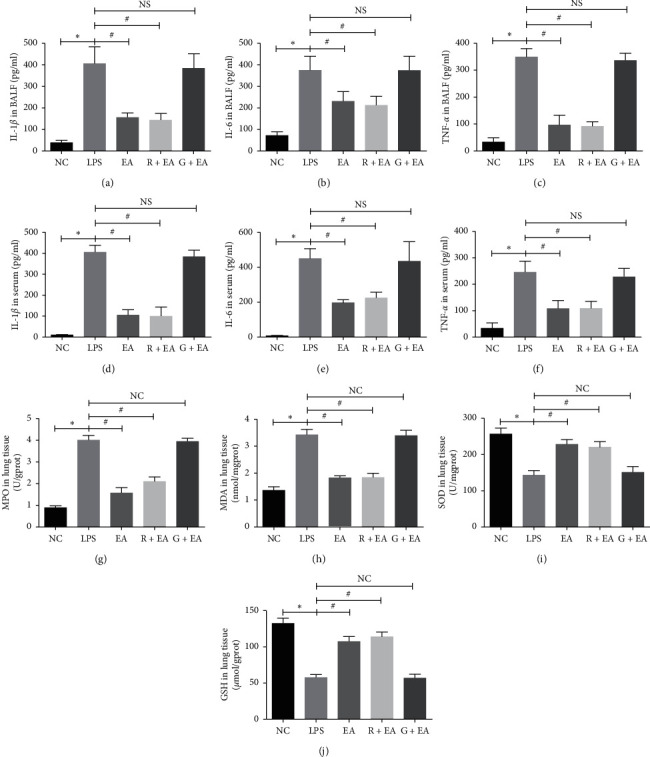
EA pretreatment suppresses the inflammatory response and oxidative stress damage. (a)–(c) Protein concentrations of IL-1*β*, IL-6, and TNF-*α* in BALF; (d)–(f) protein concentrations of IL-1*β*, IL-6, and TNF-*α* in serum; (g) the level of MPO; (h) the level of MDA; (i) the level of SOD; (j) the level of GSH. Data are presented as mean ± SEM. ^*∗*^*P* < 0.05 versus NC group, ^#^*P* < 0.05 versus LPS group.

During the process of ARDS, the inflammatory response and oxidative stress play critical roles. Thus, it is important to explore the effect of EA on the changes of inflammatory cytokines and oxidative stress markers. Levels of inflammatory cytokines in BALF and serum were detected by ELISA in all groups. As shown in Figures 3(a)–3(c), the concentrations of IL-1*β*, IL-6, and TNF-*α* in BALF of LPS group were significantly higher than those in NC group, and EA pretreatment effectively suppressed the production of these inflammatory cytokines. Rosiglitazone treatment reduced the levels of these cytokines and their expressions were almost the same as those in LPS group after GW9662 treatment. As shown in Figures 3(d)–3(f), the serum levels of these three cytokines in LPS group were increased compared with those of NC group, and EA pretreatment lowered their levels. In addition, when PPAR*γ* was activated by rosiglitazone, the expressions of the inflammatory cytokines were downregulated. The level of inflammation in G + EA group was similar to that in LPS group. MPO is highly expressed in neutrophil and the activity of MPO reflects neutrophil infiltration. In this study, MPO activity in lung tissues was elevated in LPS group and reduced in EA group (Figure 3(g)). These results suggested that EA pretreatment could effectively inhibit LPS-induced inflammatory response and this effect could be regulated by PPAR*γ*.

To clarify the protective effect of EA on oxidative stress damage, the levels of malondialdehyde (MDA), superoxide dismutase (SOD), and glutathione (GSH) were detected in the rat lung tissues. MDA was significantly elevated in LPS group compared with those of NC group and EA pretreatment group. The levels of SOD and GSH showed the opposite trend (Figures 3(h)–3(j)). These results suggested that EA pretreatment reduced inflammatory response and oxidative stress damage to prevent ARDS.

### 3.4. EA Pretreatment Inhibits Cell Apoptosis in the Rat Lung Tissues

**Figure 4:**
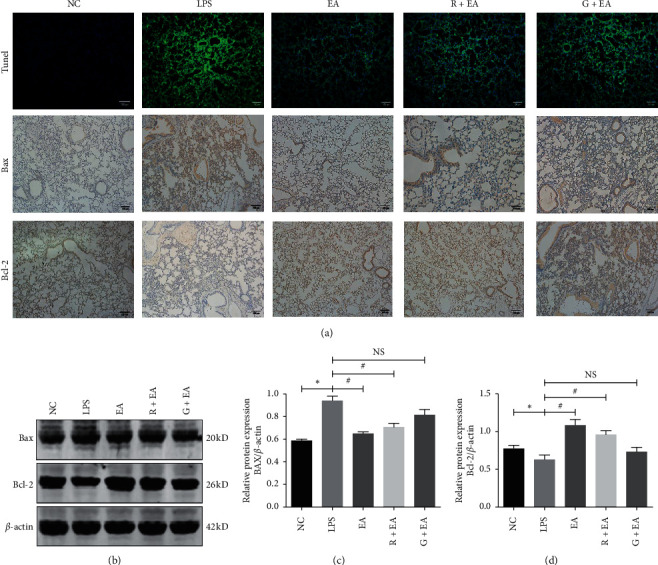
EA pretreatment inhibits cell apoptosis in the rat lung tissues. (a) TUNEL staining and immunohistochemistry analysis for Bax and Bcl-2 in the rat lung tissues (100 X). (b) Western blot analysis of Bax and Bcl-2 in the rat lung tissues. (c, d) Relative densities of Bax (c) and Bcl-2 (d). Data are presented as mean ± SEM. ^*∗*^*P* < 0.05 versus NC group, ^#^*P* < 0.05 versus LPS group.

Lung injury is known to be associated with cell apoptosis [17, 18]. TUNEL staining was used to determine the antiapoptosis property of EA (Figure 4(a)). It was found that only a very small number of positive TUNEL staining points were detected in the lung tissue in NC group. However, a great number of positive cells were seen diffusely distributed in LPS group. ARDS-induced cell apoptosis was reduced after EA pretreatment as compared with that in LPS group, showing no significant difference between EA and R + EA groups. Moreover, we further detected the expression of Bax and Bcl-2, two critical apoptosis-related proteins, by immunohistochemical staining (Figure 4(a)) and western blot analysis (Figures 4(b) and 4(c)). The immunohistochemical staining results indicated that LPS instillation increased the expression of Bax and decreased the expression of Bcl-2 when compared with NC group. In addition, when PPAR*γ* was activated by rosiglitazone, the expressions of these two proteins were similar to EA group. The levels of these two proteins in G + EA group were similar to that in LPS group. These results indicated that EA pretreatment suppressed cell apoptosis to provide a protective effect in the rat lung tissues after ARDS damage.

### 3.5. EA Pretreatment Inhibits NF-*κ*B Expression in LPS-Induced Acute Lung Injury

**Figure 5:**
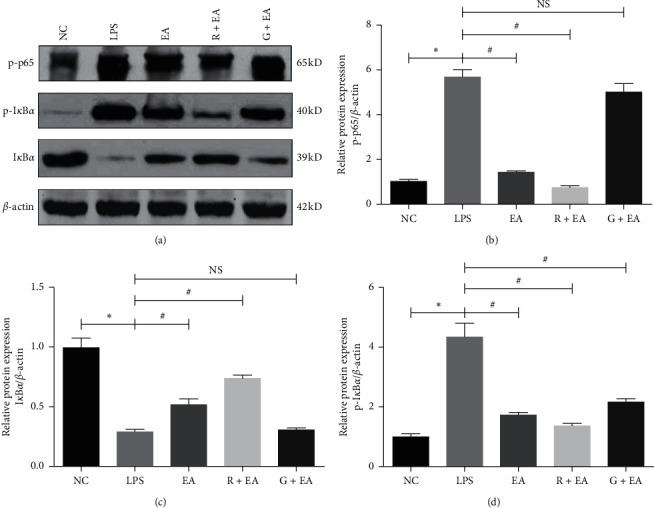
EA pretreatment inhibits NF-*κ*B expression in LPS-induced acute lung injury. (a) Western blot analysis of p-p65, p-I*κ*B*α*, and I*κ*B*α* in five groups. (b)–(d) Relative densities of p-p65, p-I*κ*B*α*, and I*κ*B*α* protein relative to that of *β*-actin. Data are presented as mean ± SEM. ^*∗*^*P* < 0.05 versus NC group, ^#^*P* < 0.05 versus LPS group.

It was known that PPAR*γ* can directly bind to the subunit p65/p50 of NF-*κ*B to form a transcriptional repressor complex [19]. This complex can reduce the binding activity of NF-*κ*B and suppress the transcription of the downstream genes. To further explore the protective mechanism of EA in ARDS, we detected the expressions of p-p65, p-I*κ*B*α*, and I*κ*B*α* via western blot analysis in different groups. As shown in Figure 5, the expression of p-p65 and p-I*κ*B*α* was upregulated significantly in LPS group when compared with that in NC group, suggesting that LPS activated the NF-*κ*B signaling pathway. However, EA pretreatment suppressed the expression levels of p-p65 and p-I*κ*B*α* and increased the expression level of I*κ*B*α*. After treatment with GW9662, the antagonist of PPAR*γ*, the expression levels of these proteins showed similar trends to that in LPS group, which indicated that the activation of NF-*κ*B signaling pathway can be regulated by PPAR*γ*. Therefore, we concluded that EA inhibited the NF-*κ*B pathway via upregulating PPAR*γ* to alleviate ARDS damage.

## 4. Discussion

In the present study, we found that EA pretreatment alleviates lung injury in rats ARDS model by reducing inflammatory response, reducing oxidative stress, and decreasing apoptosis. The underlying mechanism involves activating PPAR*γ* and inhibiting NF-*κ*B. These results suggested that EA is a potential therapy to ameliorate LPS-induced ARDS.

ARDS is a kind of respiratory diseases with high mortality, involving uncontrolled systematic inflammatory responses, ROS damage, and cell apoptosis [20]. In this study, we selected acupoint Hegu (LI4) for EA pretreatment as previously described [21] to evaluate the effect of EA in the process of ARDS. The lung histological analysis was similar to our previous study [10], and lung tissues in LPS group were represented as alveolar septal thickening, interstitial edema, and vascular congestion, all of which were relieved by EA pretreatment. Knowing that the protein content in BALF, W/D, and PaO_2_ were significantly related to the degree of lung injury [21], we detected these markers in our study and found the protein content in BALF and W/D were increased and PaO_2_ was decreased in LPS group, while EA pretreatment decreased BALF and W/D and increased PaO_2_ in EA group. In addition, we found that the effect of EA pretreatment could be reversed by PPAR*γ* antagonist GW9662, suggesting that LPS-induced ARDS could be alleviated by EA via the PPAR*γ*-dependent pathway.

It was well known that the pathophysiological process of ARDS is complex, and oxidative stress and inflammation are two major processes which cause ARDS [22]. GSH plays a protective role during the process of oxidative stress damage caused by free radicals and GSH is a good indicator of oxidative stress status and lung injury [23]. SOD catalyze the conversion of superoxide into oxygen and hydrogen peroxide and control the levels of a variety of reactive oxygen species (ROS) and reactive nitrogen species [24]. EA pretreatment alleviated the downregulated levels of GSH and SOD caused by LPS instillation, indicating that EA pretreatment alleviated the oxidative stress in LPS group. MPO is an important peroxidase enzyme to reflect neutrophil infiltration during the process of inflammation [25]. MDA is an end product of lipid peroxidation that can serve as a mediator of both oxidative stress and inflammation, commonly used as a measure of oxidative stress in biological materials [26]. In this study, MPO and MDA activities in rat lung tissue were upregulated in LPS group, and EA pretreatment reduced MPO and MDA activities.

Cell apoptosis plays a critical role in ARDS and previous study demonstrated that free radicals caused by stimulation induce Bax activation and Bcl-2 inhibition and finally cause apoptosis [27]. In this study, TUNEL staining showed the number of TUNEL-positive cells significantly higher in the rat lung tissues in LPS group. EA pretreatment decreases the number of positive cells. Moreover, the expressions of Bax increased and Bcl-2 decreased in LPS group compared with those in NC group. However, EA pretreatment downregulates the expression of Bax and upregulates the expression of Bcl-2.

The relationship between EA and PPAR*γ* has been explored in patients with Alzheimer's disease (AD) and type 2 diabetes mellitus (DM2) [8, 9] and it was reported that PPAR*γ* played a crucial role in anti-inflammatory activities [28] and activation of PPAR*γ*, which is known to be essential for the inflammation-suppressive effects, like inflammatory cytokine production and mitochondrial activities [29, 30]. However, there is no study reporting how EA affected the PPAR*γ* gene in ARDS. In our previous study [31], we found that PPAR*γ* was involved in M2 macrophage polarization, which showed a protective effect in Ali. In the current study, we also found that the expression of PPAR*γ* was decreased in LPS group and increased with the pretreatment of EA. Meanwhile, the level of inflammation in lung tissues was also opposite to the expression of PPAR*γ*. However, when PPAR*γ* was blocked by GW9662, the effect of EA was obviously decreased. These results were consistent with the possibility that PPAR*γ* downregulation had impact on ARDS and EA may have a protective effect on the injury.

Some investigators found that the anti-inflammatory and organ-protective properties of EA were associated with NF-*κ*B inactivation in some diseases such as middle cerebral artery occlusion and reperfusion (MCAO/R) [32], focal cerebral ischemia [33], DM [34], and acute kidney injury [35]. In this study, we found the expression of I*κ*B*α* was regulated by PPAR*γ*, while the use of GW9662 decreased the expression of I*κ*B*α* and increased the expression of p-I*κ*B*α*. Additionally, the expression of p-p65, a subunit of NF-*κ*B, was similar to the level of p-I*κ*B*α*. Previous studies have shown that I*κ*B*α* is the inhibitor of NF-*κ*B and increased NF-*κ*B activity can be achieved by the removal of NF-*κ*B inhibitors [36], and ubiquitous removal of NF-*κ*B inhibitors results in severe inflammation and early postnatal death [37], which were similar to our findings and the expression of the downstream of NF-*κ*B, IL-1*β*, IL-6, and TNF-*α*, which are considered to be important in regulating inflammatory response [38]. In this study, we found that the levels of IL-1*β*, IL-6, and TNF-*α* in serum and in BALF were significantly increased in LPS group and decreased in EA group. In addition, GW9662 reversed the effects of EA against LPS-induced ARDS.

Several limitations of this study should be considered. Firstly, it is reported that acupuncture has bidirectional regulation effects. In different conditions, acupuncture would play different roles [39, 40]. In order to have a better understanding of the lung protective effects of EA, it is better for us to study the effect of EA on basal lung physiology. Secondly, it is important to choose proper acupoints because acupoint specificity is an important basis for guiding traditional acupuncture practice [41]. Thus, whether EA at other acupoints can show better outcomes remains to be further explored. Thirdly, since EA and rosiglitazone are both the agonist of PPAR*γ*; it is an interesting phenomenon that there are no synergistic effects on suppressing the inflammatory response and oxidative stress damage. Thus, in our further study, we want to use PPAR*γ* knockout mice to verify the protective role of EA on ARDS.

## 5. Conclusion

In conclusion, this study suggested that EA pretreatment plays a protective role in ARDS; the mechanisms of this effect involve inhibiting inflammatory cytokines, restraining oxidative stress markers and cell apoptosis, and regulating PPAR*γ* activation and NF-*κ*B inactivation in lung tissues. The results demonstrated that EA is a potential therapy to ameliorate ARDS.

## Data Availability

The data in this study are available from the corresponding author upon request.
